# An in silico comparative transcriptome analysis identifying hub lncRNAs and mRNAs in brain metastatic small cell lung cancer (SCLC)

**DOI:** 10.1038/s41598-022-22252-7

**Published:** 2022-10-27

**Authors:** Arsham Mikaeili Namini, Motahareh Jahangir, Maryam Mohseni, Ali Asghar Kolahi, Hossein Hassanian-Moghaddam, Zeinab Mazloumi, Marzieh Motallebi, Mojgan Sheikhpour, Abolfazl Movafagh

**Affiliations:** 1 Department of Animal Biology, Faculty of Biological Sciences, Kharazmi University, Tehran, Iran; 2 Department of Cell and Molecular Biology, Faculty of Life Sciences and Biotechnology, Shahid Beheshti University, Tehran, Iran; 3 Department of Social Medicine, School of Medicine, Shahid Beheshti University of Medical Sciences, Tehran, Iran; 4 Social Determinants of Health Research Center, Shahid Beheshti University of Medical Sciences, Tehran, Iran; 5 Department of Biology, Zanjan Branch, Islamic Azad University, Zanjan, Iran; 6 Department of Medical Genetics, School of Medicine, Shahid Beheshti University of Medical Sciences, Tehran, Iran; 7 Department of Mycobacteriology and Pulmonary Research, Pasteur Institute of Iran, Microbiology Research Center (MRC), Pasteur Institute of Iran, Tehran, Iran

**Keywords:** Cancer, Computational biology and bioinformatics, Genetics

## Abstract

Small cell lung cancer (SCLC) is a particularly lethal subtype of lung cancer. Metastatic lung tumours lead to most deaths from lung cancer. Predicting and preventing tumour metastasis is crucially essential for patient survivability. Hence, in the current study, we focused on a comprehensive analysis of lung cancer patients' differentially expressed genes (DEGs) on brain metastasis cell lines. DEGs are analysed through KEGG and GO databases for the most critical biological processes and pathways for enriched DEGs. Additionally, we performed protein–protein interaction (PPI), GeneMANIA, and Kaplan–Meier survival analyses on our DEGs. This article focused on mRNA and lncRNA DEGs for LC patients with brain metastasis and underlying molecular mechanisms. The expression data was gathered from the Gene Expression Omnibus database (GSE161968). We demonstrate that 30 distinct genes are up-expressed in brain metastatic SCLC patients, and 31 genes are down-expressed. All our analyses show that these genes are involved in metastatic SCLC. PPI analysis revealed two hub genes (*CAT* and *APP*). The results of this article present three lncRNAs, Including XLOC_l2_000941, LOC100507481, and XLOC_l2_007062, also notable mRNAs, have a close relation with brain metastasis in lung cancer and may have a role in the epithelial-mesenchymal transition (EMT) in tumour cells.

## Introduction

Lung cancer (LC) accounts for the most cancer-related death globally. In 2020, nearly 2.21 million patients were diagnosed with lung cancer, and 1.80 million deaths were estimated to be related to lung cancer (https://gco.iarc.fr)^1^. The Brain is the most distant metastasis in small-cell lung cancer (SCLC) patients, and brain metastasis (BM) increases to 50% for patients who survive after two years of initial diagnosis^2^; therefore, the overall survival of 5 years is 1–5%^3^. An early lung cancer diagnosis is crucial since surgery is ineffective in the late stages, especially in patients with metastasis. SCLC is an aggressive type of LC with the early development of widespread metastases; this leads to SCLC with a tremendously low prognosis for patients with the disease^4^. SCLC is highly relevant to smoking, with only 2% of patients diagnosed with this subtype never smoking in their lifetime^5^. *Rb1* and *Trp53* are the most genetic inactivation forces for SCLC; however, the most efficient genomic alterations remain unclear at^6,7^. Brain metastases occur in approximately 20% of all cancer patients, with most cases in people with lung, breast, and colorectal malignancies, melanoma, or renal cell carcinoma^8–11^. Brain metastases (BMs) were found in more than 25% of SCLC patients, with an average survival of 9 months following total therapy.

However, as imaging methods and systemic medicines advance, the increased incidence of BMs increases year after year as patients live longer. In individuals with advanced cancer, the presence of BMs continues to significantly contribute to overall cancer mortality^12^. Although more initial disease stage shift is a great result, changing the early stages of the disease is a significant result; the leading indicator of the effectiveness of screening is the reduction of mortality^13^. It is now clear that most parts of the human genome are non-coding. Long non-coding RNA (lncRNA) is the common term for transcriptomes with 200 or more nucleotides. About a decade ago, investigators figured out that lncRNAs play an essential role in cancer biology^14–17^. Dysregulation of lncRNA is the most critical evidence in different cancer types.

For instance, lncRNA *HOXB-AS3* can exacerbate lung cancer migration, proliferation, and invasion. The mechanisms underlying these changes are by activating the PI3K‐AKT pathway. The PI3K/Akt/mTOR pathway impacts tumorigenesis and cancer progression^18–20^. This breakthrough helps us to propose a potential biomarker for cancer predictivity. This study aimed to detect genes (mRNAs and lncRNAs) differentially expressed between SCLC patients with brain metastasis and SCLC patients without brain metastasis. We also performed an enrichment analysis to find correlations between specific mRNAs and lncRNAs of our two cancer patient groups. However, considering a gene as a biomarker requires deep, long, precise, and comprehensive studies.

## Methods

### Data predation and analysis

Data for lncRNA and mRNA microchip array extracted from peripheral blood mononuclear cells of SCLC patients were downloaded through Gene Expression Omnibus (GEO)^21^. The data was designed as brain metastasis (BM) patients compared to non-BM patients for lncRNAs in relation to BM in LC samples^2^ (Accession: GSE161968, Platform: GPL20115).

All six replicates of the samples were used for multiple analyses. Statistical analyses were performed in R (*v3.5.1*). All the scripts and algorithms were uploaded to the GitHub webserver.

**Figure 1:**
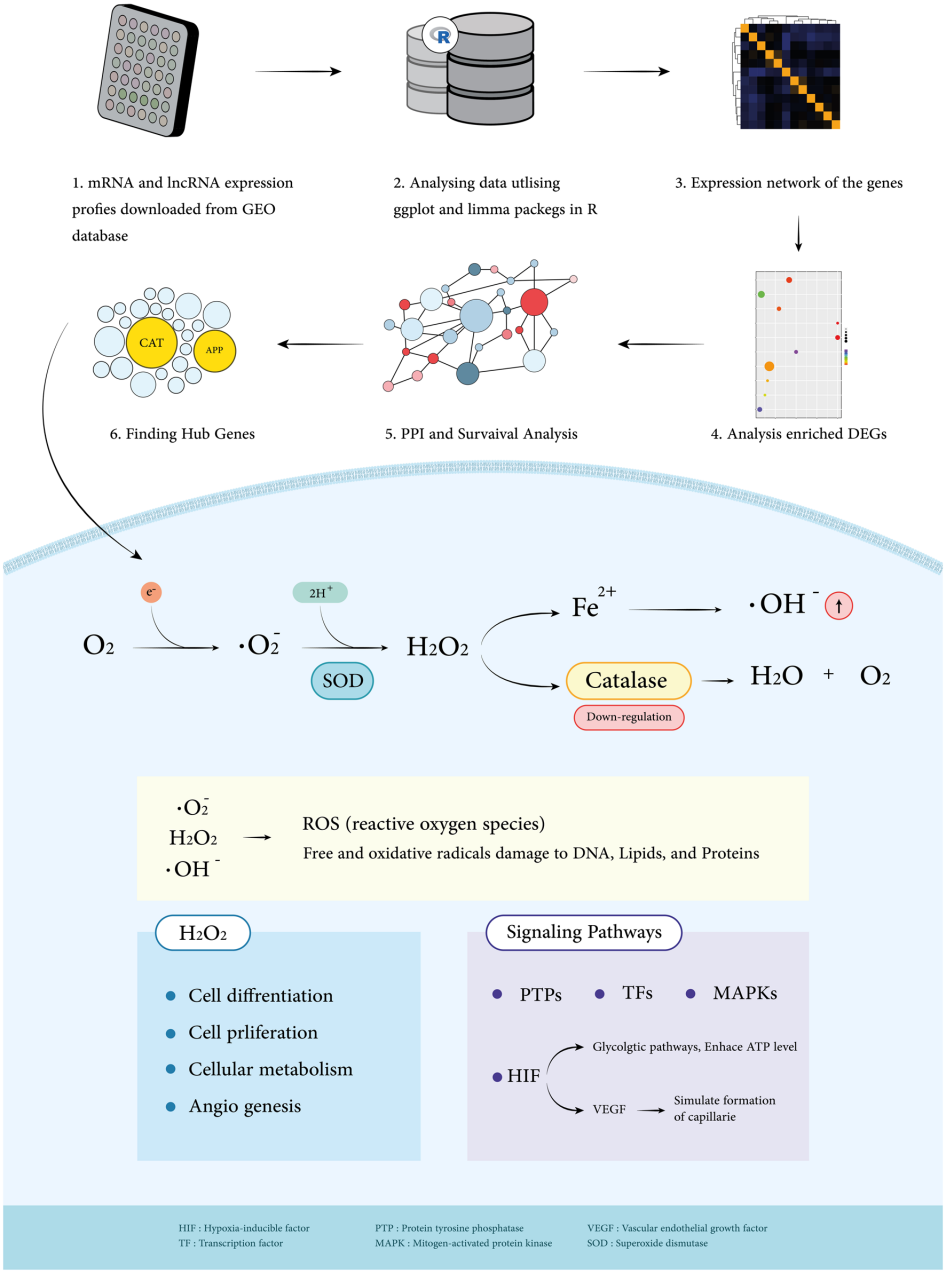
Article section workflow. Workflow represents a comprehensive review summarising this study's data gathering, trimming, and analysing approaches. *CAT* (catalase), for instance, is one down-regulated hub gene that shows significant roles in critical pathways.

After constructing a tidy data set of gene expression matrix with samples and probes IDs, we analysed the mean expression and probable correlations through boxplot and heatmap to ensure denormalisation and prevent any false correlation. Afterwards, by scaling the expression matrix on the mean expression of each gene, differentially expressed genes (DEGs) among samples arise in a MA plot. Moreover, principle component analysis was performed on our data sets' samples. Clusters were trimmed by ignoring six samples from both BM and non-BM groups. Subsequently, we manipulate the K-mean algorithm for re-clustering our data set for a strong validation. Limma package (*v3.52.2*) was used to calculate the log2foldchange (LogFC) parameter threshold to benefit the highest number of expression data well^22^. Hence LogFC ≥ 1.75 and LogFC ≤ -1.75 were applied to our DEG results as up and down-regulated genes, respectively. Genes with a *P*-value of ≤ 0.05 were considered significant. We consider 1.75 for log2foldchange as our cutoff value because a number outside this range would give us an inappropriate number of candidates. Alternatively, genes with higher expression in BM than non-BM samples were categorised as up-regulated and down-regulated. A workflow of our approaches in this study is provided (Fig. 1).

### Enrichment analysis

Understanding the underlying biological roles of various genes or proteins is best accomplished through Gene-Set Enrichment. Through enrichment analysis, the interpretability of biological insights is enhanced, and the complexity of molecular data is reduced^23^. The basic idea of Gene Set Enrichment Analysis (GESA) is to explore groups of genes considerably over-represented in a given list of genes compared to a control set of genes. These gene sets are often, but not always, made up of genes that collaborate in a recognised biological pathway^24^. Enrichr (https://maayanlab.cloud/Enrichr/), a comprehensive resource for curated gene sets and a search engine provided by Maayan Megalab^25^, was applied, with results extracted from various databases for enrichment analysis. After exporting the results of our gene dataset, the *P*-value was set to ≤ 0.05.

CBioPortal (https://www.cbioportal.org/) is a database that provides visualisation, analysis, and large-scale cancer genomics data sets^26^. It is a tool for the interactive examination of multidimensional cancer genomics data sets, which was first created at Memorial Sloan Kettering Cancer Center^27^. This database was used to find mutations in the candidate genes; then, all small cell lung cancer datasets were selected, including Small Cell Lung Cancer CLCGP (Nat Genet 2012), Johns Hopkins (Nat Genet 2012), U Cologne (Nature 2015), and Multi-Institute (Cancer Cell 2017). Abundance and types of mutations for these genes were found.

### Gene and protein network analysis

GeneMANIA (http://genemania.org/) plugin in Cytoscape (https://cytoscape.org/) version 3.9.1 was applied to show the relations between candidate up and down-regulated genes. GeneMANIA is a user-friendly online service that allows the generation of gene function hypotheses, analysis of gene lists, and prioritise genes for functional experiments. GeneMANIA will automatically weight data sources based on their predictive value for rebuilding the query list, provided that the list is big enough (currently five or more genes). For instance, GeneMANIA will heavily weigh protein domain similarity networks and recommend more genes with a similar domain structure when provided with query genes with comparable protein domain structures. GeneMANIA frequently discovers new members of a protein complex based on query genes that are a part of that complex and gives high weight to actual or anticipated physical interactions^28^. Cytoscape is a free, open-source software project that combines biomolecular interaction networks, high-throughput expression data, and other molecular states into a cohesive conceptual framework.

Protein–protein interactions (PPI) also were performed from the String protein database plugin (https://string-db.org/) using Cytoscape to indicate networks of proteins of these genes. The STRING database attempts to combine all known and expected protein–protein interactions, including physical and functional ones^29^. For the past 19 years, the STRING database has been in steady expansion, and the current edition includes protein interaction data for approximately 2000 species; nonetheless, all interactions are strictly intra-species. For the first time, we incorporate cross-species interactions in the STRING database in this study. STRING reported that PPIs are functional connections between proteins. These connections are not restricted to physical interactions; they may involve interactions like transcription factor binding or represent that the linked proteins are found in the same biological pathway^30^.

Panther (Protein Analysis Through Evolutionary Relationships) (http://www.pantherdb.org/) classification systems were applied to classify our candidate protein-coding genes. PANTHER is a comprehensive knowledge base of evolutionary and functional relationships between protein-coding genes (often referred to as proteins) and tools for utilising the classifications to analyse large-scale genomics data. Evolutionary groupings (Protein Class, Protein Family, Subfamily) and functional groupings are the main categories used to classify proteins (Gene Ontology and pathways)^31^. The type of protein classes and their percentage were obtained.

### Survival analysis

We employed the Kaplan–Meier plotter database (https://kmplot.com/analysis/) to perform a survival analysis of the hub genes. It is a web-based tool that allows for pooled survival analysis by combining different datasets from the cancer Biomedical Informatics Grid (caBIG), GEO, and TCGA repositories^32^. Up to April 24th, 2022, this database presented the overall survival analysis data of 1925 lung cancer patients. We input our hub genes separately and consider a *p*-value threshold of 0.05 for a meaningful result. Although KM-Plotter has many filters for focusing the study on specific criteria like stage, gender, age, surgery success, histology, chemotherapy, and radiotherapy, we performed the analysis on default restrictions, making our results more comprehensive and general.

## Results

After DEGs evaluation, we need to understand molecular mechanisms that would be affected by these genomic dysregulations and how the disturbances impact them. DEGs in SCLC BM and non-BM patients are related to transcription factors, non-coding RNAs, mutations, and signaling pathways. To understand the expression patterns in our gene dataset, we candidate 30 up-regulated and 31 down-regulated genes, including mRNAs and lncRNAs, in BM and non-BM patients (Table 1). The relations and overlap of our candidate genes with transcription factors, miRNAs, histone modifications, signalling pathways, cell types and tissues, and Top 10 correlated diseases acquired from specific databases for each group. (See Tables S2, S3, S4, S7, S8, and S9). In addition, BM shows different protein classes for up-regulated (16 protein-coding genes) and down-regulated (14 protein-coding genes) in BM, as shown in Fig. 6. Each gene with its class protein is demonstrated In Tables S5 and S6 for up and down genes.

**Table 1:** Candidate genes with different expressions in brain metastasis patients with SCLC. *p*-value ≤ 0.05.

Up-regulate genes	logFC	*P*-value	Down-regulate genes	logFC	*P*-value
PRSS33	3.383007303	0.004560695	OCLN	− 3.566468452	0.022160353
WHAMMP2	2.99733975	0.005815325	XLOC_l2_007062	− 3.329664096	0.013558958
XLOC_l2_000941	2.687330175	4.1957237415278E-05	ZIM2	− 2.854825242	0.028619212
MYOM2	2.601569126	0.021263756	PNMA2	− 2.660709775	0.01010599
C1orf87	2.59361413	1.62961553882741E-05	MPO	− 2.603630721	0.034713463
PIGC	2.568682566	0.04592453	ANKRD34B	− 2.435841569	0.049785848
C1orf105	2.537416642	0.004661527	ABCA13	− 2.425532635	0.010254371
ALOX15	2.372469038	0.006367303	LOC646627	− 2.409993098	0.037554779
IGIP	2.276075691	0.000645279	SLC14A1	− 2.380643274	0.001828896
SPAG6	2.242164253	0.025026256	ITLN1	− 2.341855873	0.003294184
LPAR3	2.233936235	0.019032771	CRB1	− 2.310587746	0.014682181
ZNF416	2.102556319	0.000160004	PGK2.00	− 2.111562308	0.019712849
EMR4P	2.088110399	0.006628096	KRT1	− 2.043848253	0.023066534
TRIP13	2.06727697	0.000330399	SLC2A5	− 1.999212172	0.031409788
IL5RA	2.061453481	0.006381159	PGLYRP1	− 1.943771474	0.032295719
TM4SF19	2.012294101	0.005361822	FAM83A	− 1.926935845	0.002982599
PCDHGA9	2.009416266	0.00140228	XK	− 1.915279305	0.008257932
PIGW	1.995038208	0.038464331	DPCD	− 1.83374777	0.040212628
ZNF599	1.985801014	0.001438597	AMFR	− 1.828192394	0.000293755
LGSN	1.972203834	0.006590149	TMEM158	− 1.802895011	0.001143757
TMEM176B	1.953156305	0.023746468	DACH2	− 1.800987665	0.00095326
TMEM176A	1.935063753	0.030140607	OSBP2	− 1.790583828	0.044198947
TTC39B	1.907722674	0.014295311	CRAT	− 1.788273793	0.012034785
SIGLEC8	1.861465376	0.004428622	ZNF608	− 1.78743558	0.002590702
PRAMEF20	1.856405961	0.02697871	CYBRD1	− 1.776635568	0.001412057
CLC	1.853046847	0.010293129	CTSB	− 1.776261192	0.012873076
EGR3	1.843233413	0.003359614	ANXA3	− 1.767371637	0.010699241
LOC100507481	1.77545701	0.001358476	RIOK3	− 1.762783691	0.006681225
ZNF827	1.764674234	0.01536046	MAOB	− 1.758125128	0.00024905
HSD17B3	1.757453949	0.0062133	LOC729870	− 1.757276671	0.035413402
			IFT52	− 1.752051545	0.014110306

### Non-coding RNAs might be directly related to brain metastasis in SCLC

Enrichment analysis displayed that our candidate genes are involved in essential cell signalling pathways. Some of these genes are important enough that we bring them up in the discussion section, such as *IL5RA*, *EGR3*, and *ALOX15* as up-expressed genes, and *OCLN*, *MAOB*, *CTSB*, and *MPO* as down-expressed genes in BM SCLC. In the category of up-regulated genes, we find LOC100507481 and XLOC_l2_000941 as lncRNAs; *WHAMMP2*, as a pseudogene encodes 5246 nucleotide transcript length; *ADGRE4P*, also a pseudogene encodes 2732 nucleotide transcript length; *TTC39B* is a protein-coding but encodes non-coding RNA with 7023 nucleotide transcript length; *ZNF827* another protein-coding that encodes 3675 nucleotide transcript and *HSD17B3* protein-coding genes that encode a 3559 nucleotide transcript length. For down-regulated genes, we find XLOC_l2_007062 and LOC729870 as a lncRNA; *ZIM2* is a protein-coding gene that encodes four non-coding RNA with approximately 2300 nucleotide transcripts length; Similar to this, *ABCA13* also is a protein-coding gene that encodes five non-coding RNA with approximately 15,000 nucleotide transcripts length; *ZNF608* encodes non-coding RNA about 6000 nucleotides, *ANXA3* encodes 1056 nucleotide transcripts and *RIOK3* about 3500 nucleotides, also are protein-coding genes. Our ultimate goal is to demonstrate the lncRNAs related to BM in SCLC patients. To assess our targeted lncRNAs, we utilised lncHUB, Gene Ontology (GO), and Kyoto Encyclopedia of Genes and Genomes (KEGG) pathway enrichment analysis and trimmed our dataset based on *P*-Value (*p* < 0.05)^33–35^. Since our microarray data was performed on lncRNAs and mRNAs, we utilised multiple databases to discover the correlated lncRNA and mRNAs to conduct a comprehensive study on pathways and biosystems with relatively close lung cancer brain metastasis.

### Investigation of gene ontology and pathways

In order to investigate candidate genes' functions in the cell, it is essential to show their signalling pathways to find their role in cancer; The KEGG database is required to achieve this aim. KEGG (2021) pathway enrichment analysis showed three significant genes; *ALOX15*, *PIGC*, and *PIGW*, which correlated in Ferroptosis and Linoleic acid metabolism pathways and Glycosylphosphatidylinositol (GPI)-anchor biosynthesis. (Table S1 shows the result of the KEGG analysis). Gene ontology (GO) enrichment analysis is commonly used to assign functions to gene sets and obtain insights into the biological processes in which they are engaged^31^. GO is a collection of gene function-controlled vocabularies arranged into three ontologies: biological process, cellular component, and molecular function. Due to community initiatives, many genomes have been annotated with GO keywords, allowing GO enrichment analysis on multiple gene sets. An enrichment study, for example, identifies GO keywords that are over-represented for a specific collection of human genes, allowing for biological interpretation.

**Figure 2:**
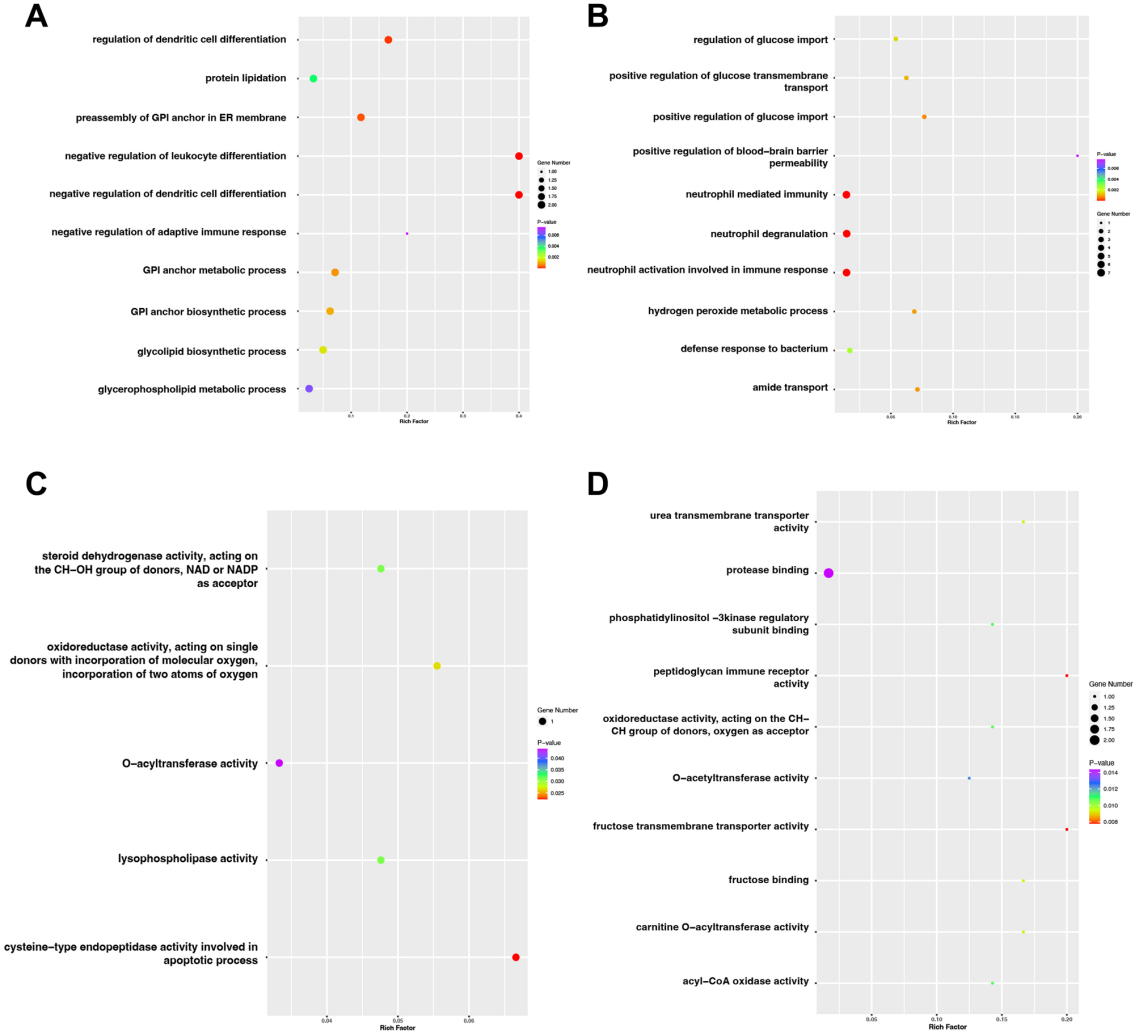
Gene ontology (GO) enrichment analysis. (**A**) Top 10 most significant changes in the GO biological process for up-regulated genes. (**B**) Top 10 most significant changes in the GO biological process for down-regulated genes. (**C**) Top 5 most significant changes in the GO molecular functions for up-regulated genes. (**D**) Top 10 most significant changes in the GO molecular functions for down-regulated genes.

On the other hand, KEGG pathway mapping provides more information about how genes or gene products interact in pathways, although the coverage of genes is less extensive than GO. GO (2021) enrich analysis was performed on our candidate genes from DEGs results. The top five GO terms with desirable *P*-Value and Combined Scores are listed in Table S1. Moreover, among all GO databases results on our DEGs, the top 10 molecular function and biological process terms of both up and down-regulated genes are due to the rich factor of each term shown in Fig. 2. We utilise ggplot2 (*v3.3.6*) to visualise critical biological processes^36^.

### Pathway and cell type and tissue analysis

Pathways influenced by these candidate genes are briefly explained in Tables S2 and S3; *SLC14A1*, *SLC2A5, MAOB*, *CRAT*, *ANXA3* down genes; *PIGC, PIGW, LPAR3, IL5RA, HSD17B3, EGR3* up genes, involved in different essential pathways. Our study focuses on brain metastasis; hence in table S4, Key cell types and tissues related to up and down-regulated genes in brain metastasis patients demonstrated, as *TMEM176B*, *ALOX15*, *TMEM176A* up and *SLC14A1*, *OSBP2 XK*, *KRT1* down genes are significant. At last, for comprehensive analysis, some other diseases related to brain metastasis SCLC genes and SCLC without brain metastasis genes are presented in Table S9 that *LPAR3*, *TTC39B*, *TMEM176B*, *IL5RA* up expressed genes and *ZNF608*, *DACH2*, *ZIM2* down expressed, are related.

### *PIGW*, a critical lncRNA

After analysing our candidate's genes, we monitor *IGIP*; *PCDHGA9*, *TMEM176B*; *TMEM176A* are up expressed and *OCLN*; *CRB1*; *PNMA2*, *ITLN1*, and *ANXA3* are down-expressed genes (shown in Table 1) has correlations with noticeable lncRNAs. For example, *PIGW* observed concerning multiple pathways in previous analysis has a co-expression relation with LINC01386 and might have a critical molecular mechanism in brain metastasis in lung cancer. To survey key lncRNAs related to candidate genes, we utilised an enrichment analysis tool, which has presumed a constant *p*-value affiliated with input genes and its datasets. lncRNA co-expression analysis performed on the lncHUB database for both up and down-regulated genes, and the *p*-values equal 0.00983 for up-regulated genes and 0.010472 for down-regulated genes, respectively (Table 2)^37^.

**Table 2:** Key LncRNAs related to up and down-regulated genes in brain metastasis patients (lncHUB lncRNA Co-Expression database).

LncRNA	Up-regulated genes	LncRNA	Up-regulated genes	LncRNA	Down-regulated genes
BDNF-AS	IGIP; PCDHGA9	LINC02712	SIGLEC8; PCDHGA9	FGF10-AS1	AMFR; CYBRD1
RASSF8-AS1	IGIP; PCDHGA9	LINC00501	TMEM176B; TMEM176A	LINC02115	AMFR; CYBRD1
TMEM161B-AS1	IGIP; PCDHGA9	LACTB2-AS1	TMEM176B; TMEM176A	LINC01716	AMFR; ZIM2
NNT-AS1	IGIP; PCDHGA9	C1RL-AS1	TMEM176B; TMEM176A	MYO16-AS1	ANXA3; FAM83A
SEPTIN7-AS1	IGIP; PCDHGA9	LINC02068	TMEM176B; TMEM176A	LINC00424	ANXA3; ITLN1
PSMG3-AS1	IGIP; PCDHGA9	LINC01853	TMEM176B; TMEM176A	LINC00560	ANXA3; ITLN1
BACE1-AS	IGIP; PCDHGA9	LINC01504	TMEM176B; TMEM176A	CTNNA2-AS1	CRB1; PNMA2
GRID1-AS1	IGIP; PCDHGA9	FBXO3-DT	TMEM176B; TMEM176A	LINC01114	CRB1; PNMA2
LINC00909	IGIP; PCDHGA9	LINC01843	TMEM176B; TMEM176A	LINC02520	CRB1; ZIM2
LINC01511	LGSN; MYOM2	LINC02384	TMEM176B; TMEM176A	LINC01701	FAM83A; CTSB
LINC02864	LGSN; TRIP13	OVOL1-AS1	TMEM176B; TMEM176A	LINC01288	MAOB; ZIM2
LPP-AS2	LPAR3; ZNF599	SMIM2-AS1	TMEM176B; TMEM176A	ARHGEF2-AS1	OCLN; ANXA3
TMCO1-AS1	PIGC; ZNF599	LBX2-AS1	TMEM176B; TMEM176A	NKX2-1-AS1	OCLN; CTSB
LINC00448	PRAMEF20; MYOM2	CPNE8-AS1	TMEM176B; TMEM176A	LINC01806	OCLN; CTSB
LINC01736	SIGLEC8; PCDHGA9	ERVK9-11	TMEM176B; TMEM176A	SCN1A-AS1	PNMA2; ABCA13
LINC02015	TMEM176B; TMEM176A	LINC02712	SIGLEC8; PCDHGA9	SAP30-DT	PNMA2; SLC2A5
LINC01880	TMEM176B; TMEM176A	LINC01999	TMEM176B; TMEM176A	HHLA3-AS1	XK; ITLN1
LINC02244	TMEM176B; TMEM176A	LINC02538	TMEM176B; TMEM176A	LINC02703	ZNF608; SLC2A5
LINC02011	TMEM176B; TMEM176A	HNF1A-AS1	TMEM176B; TMEM176A		
SMIM2-IT1	TMEM176B; TMEM176A	LINC01386	TRIP13; PIGW		
LINC01781	TMEM176B; TMEM176A	SMAD9-IT1	ZNF827; IGIP		
LINC01506	TMEM176B; TMEM176A	EDNRB-AS1	ZNF827; PCDHGA9		
LINC02532	TMEM176B; TMEM176A				

### Gene network and PPI analysis

**Figure 3:**
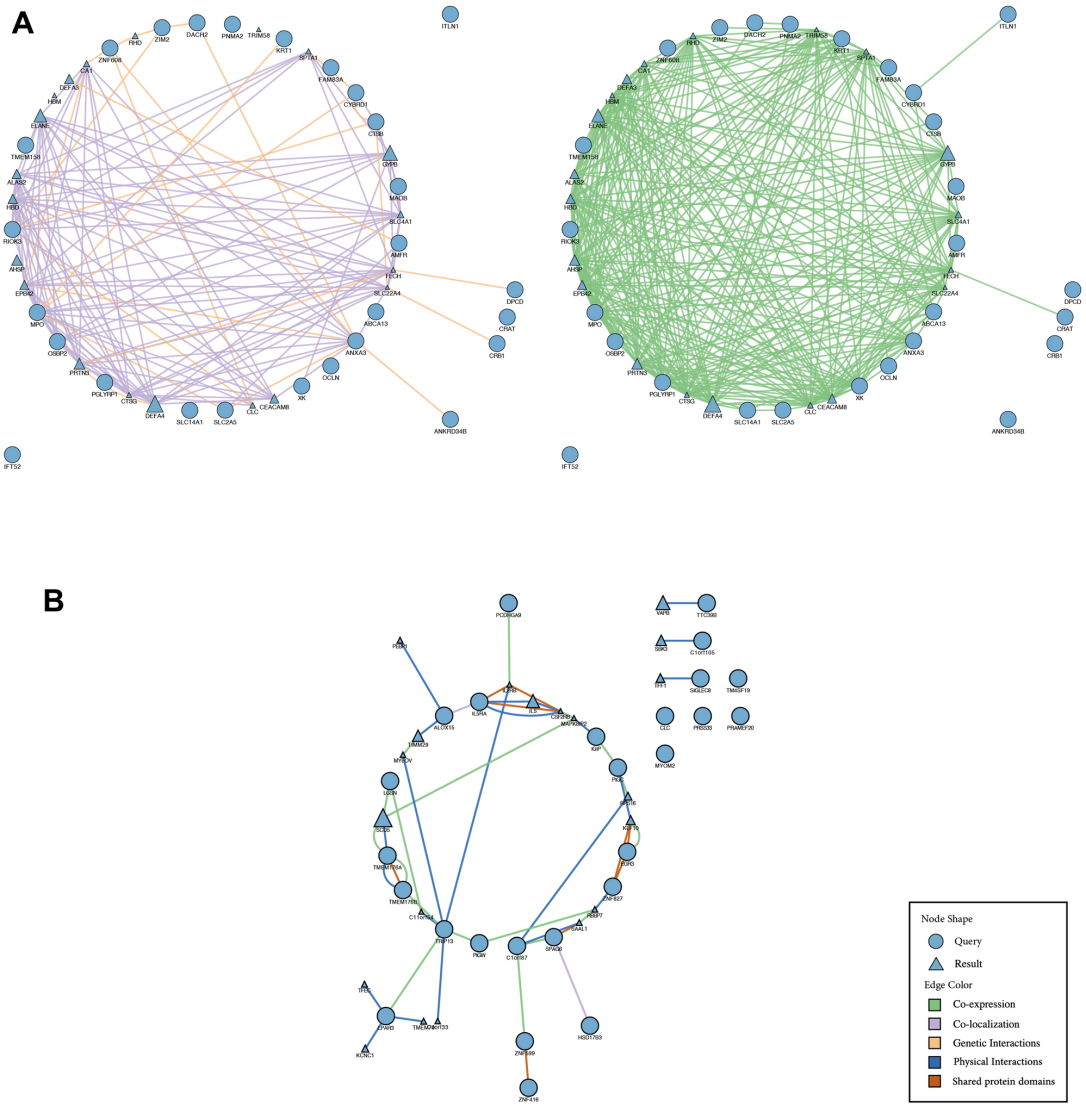
Candidate genes in BM SCLC patients—gene networks from GeneMANIA. (**A**) Up-regulated genes network. Physical interactions are shown in blue lines, Co-expression genes are shown in green lines, Co-localization is shown in purple lines and shared protein domains are shown in the orange lines. The higher scores are shown as a larger circle. Intermediate genes involved were also demonstrated. (**B**) Down-regulated genes network. Co-expression genes are shown in green lines, Co-localization is shown in purple lines, and genetic interactions are shown in orange lines. The higher scores are shown in black as a larger circle. Intermediate genes involved were also demonstrated.

Gene network analysis co-expression and co-localisation between up and down-regulated genes were performed and provided in Fig. 3.

**Figure 4:**
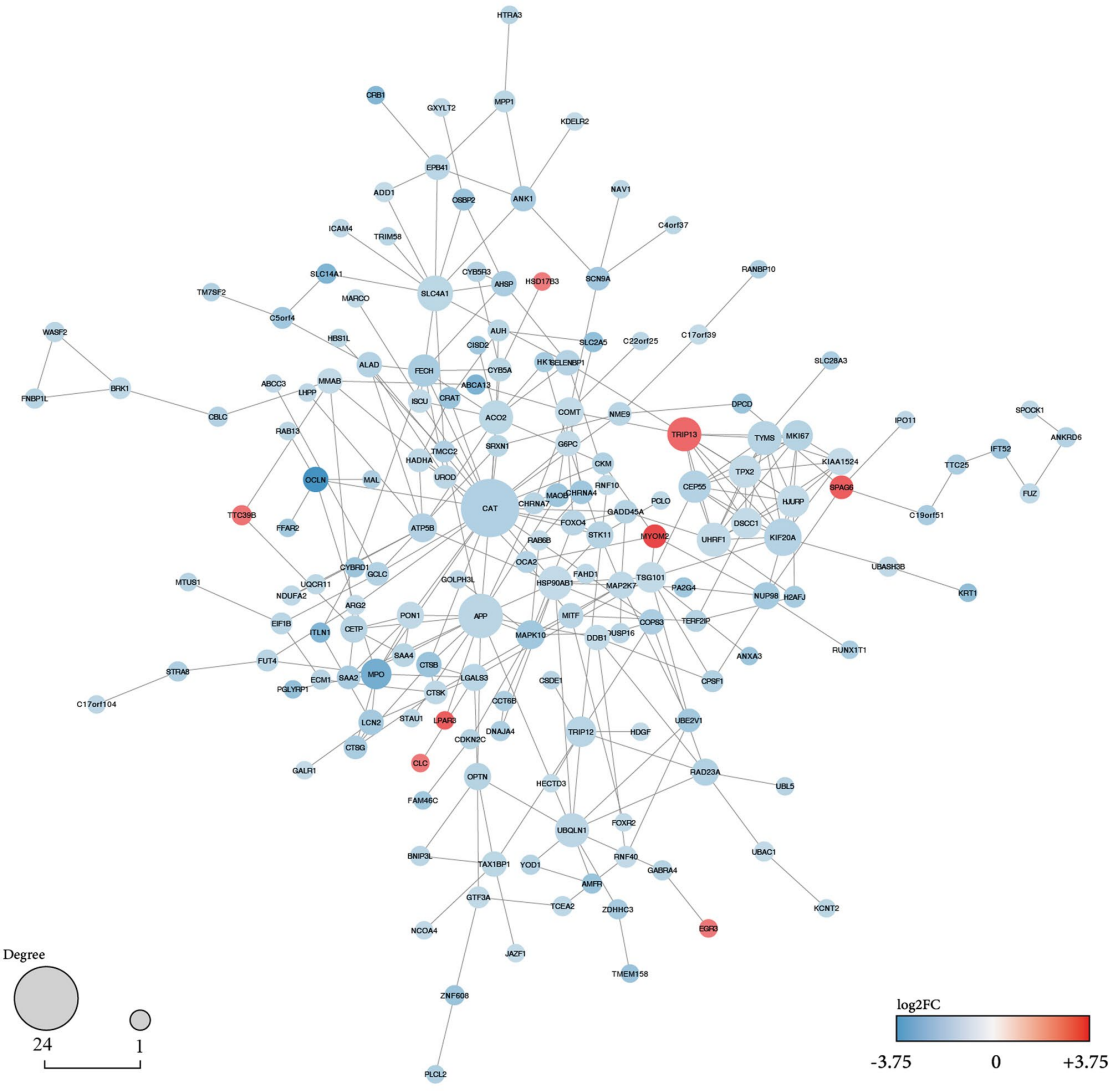
Protein–protein interactions network with DEGs for brain metastasis in SCLC. Up and down-regulated candidate genes are coloured on the scale of logFC. Log2FC is an indicator of the expression level of these genes since logFC = 3.75 for the most up-regulated gene in BM SCLC patients and logFC = − 3.75 for the most down-regulated gene. The nodes' size characterises the connectivity degree for identifying the critical hub genes (*CAT* and *APP*). Unconnected nodes are trimmed from the figure.

**Figure 5:**
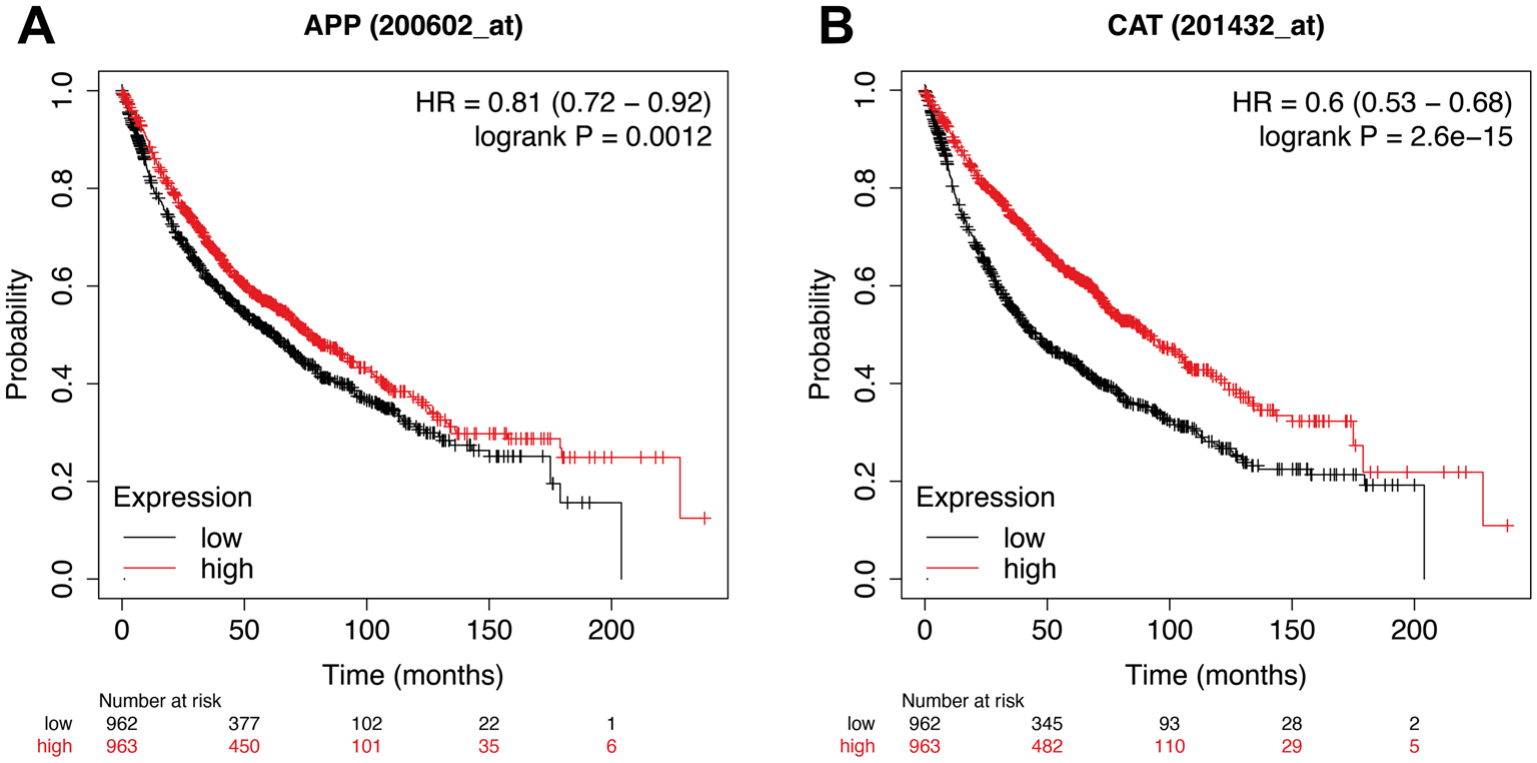
Prognostic values of two hub genes in the brain metastases from SCLC patients. Kaplan–Meier survival analysis of 2 hub genes in patients with small-cell lung cancer. The prognostic value of (**A**) *APP* and (**B**) *CAT* for determining the prognostic value of *APP* and *CAT* mRNA expression were evaluated using the Kaplan–Meier plotter. The Affymetrix IDs of the genes are as follows: 200602_at (*APP*) and 201432_at (*CAT*). Logrank test for Kaplan–Meier analysis reveals the *p*-value for both *APP* and *CAT* survival analysis. *APP* and *CAT* were specifically represented among lung metastasis samples. The patients survive probability with a high expression of hub genes indicated as red-line, and patients survive probability with a low expression of hub genes indicated as black-line. The high and low-expression cohorts were divided by the median survival time.

We used the STRING plugin in Cytoscape (version:3.9.1) for protein–protein interactions to construct a network between up and down-regulated genes according to our DEGs results^38,39^. For complete results, we filter our DEGs to |log2FC|> 1. Hence, the resulting graph showed us 169 nodes and 300 edges. Additionally, the node's colour is illustrated by log2fold change. Utilising the CentiScaPe plugin, every node's degree greater than 15 is considered hub genes, two genes (*CAT*:24 and *APP*:16)^40^ (Fig. 4). We managed to study our hub geans survival analysis (Fig. 5).

We discovered that 20% of BM patients have mutations in 28 up-regulated genes in 47 individuals in 249 samples. Figure S1 depicts this. *MYOM2* contains the most modifications (6%). This gene has mutations in I-set: Immunoglobulin I-set domains and Ig_2: Immunoglobulin domain.

In 77 cases, 32% of those queried had mutations in 27 down-regulated genes. Further analysis showed *MPO* had the most significant mutations (2.5%) in SCLC patient samples indicated in Figure S2.

### *CAT* and *ACC* are significant genes in lung cancer patients' survival

As we mentioned before, *CAT* and *ACC* are considered hub genes. Initial KP-Plotter analysis showed that the results were meaningful due to top values. The overall survival plots are provided in Fig. 5. *CAT* and *ACC*'s hazard ratio (HR) were 0.6 and 0.81, respectively. HR < 1 means that the up-regulation of genes drives high survival probability in LC patients. Our hub genes have a negative log2fold change in brain metastasis SCLC patients making these two potential biomarker genes. Fortunately, there is a meaningful relationship between DEGs and KP-Plots, validating our prediction. The hub genes *CAT* (HR = 0.6, *P*-value = 2.6e − 15, log2FC = − 1.3802) and *APP* (HR = 0.81, *P*-value = 0.0012, log2FC = − 1.1716) were significantly associated with an unfavorable overall survival in lung cancer patients.

**Figure 6:**
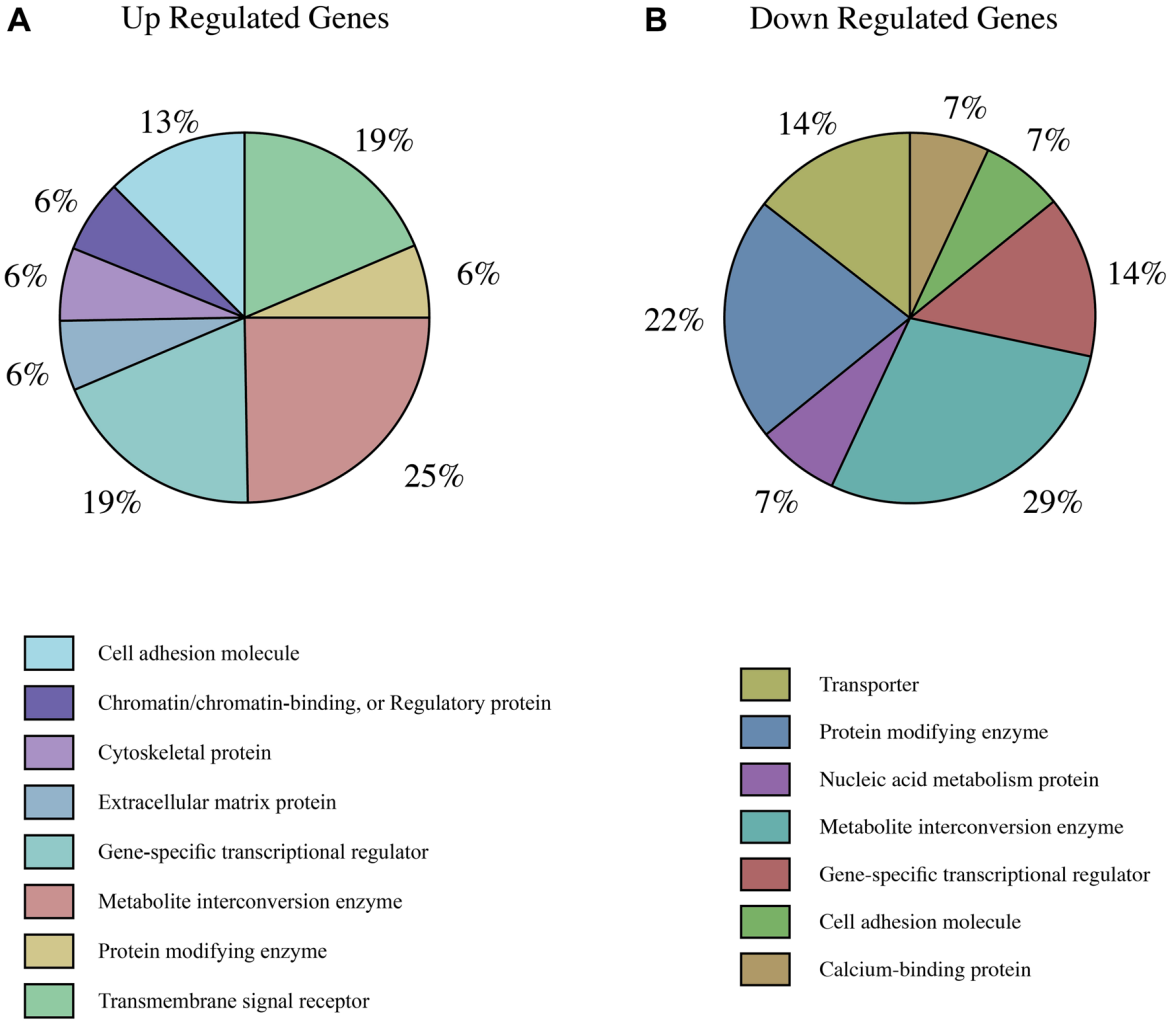
Protein classes for candidate genes in BM SCLC patients. Different categories of up and down-regulated genes are shown in (**A** and **B**), respectively.

Our candidate proteins' coding genes are involved in constructing metabolic interconversion enzymes, transmembrane signal receptors, cell-adhesion molecules, protein-modifying enzymes and gene-specific transcriptional regulators, as shown in Fig. 6. Knowing the class of proteins can help us to explore the differences between BM and non-BM lung cancer patients.

## Discussion

Many lncRNAs showed in epigenetic changes like gene imprinting, modifications, and regulations. Beyond lncRNA, there is yet another type of non-coding RNA with much less information associated with cancer biology. Small-non-coding RNA (snoRNA) has 60 to 300 nucleotides. Recent studies have shown the migration and progression of lung cancer tumours mainly through EMT and MMPs regulated by lncRNAs. Additionally, the immune system has cytotoxic CD8 + T cells to restrict tumour cell growth in cancers. Furthermore, overexpression of tumour-promoting lncRNAs can manage immune evasion of LC cells^41^. However, this paper will focus on cancer-related lncRNAs and mRNAs.

XLOC_l2_000941 third up-regulated lncRNA gene in DEGs results, with log FC = 2.68 (Table 1), is related to MYC transcription factor (TF) based on Peng-ran Sun et al. studies^42^. MYC, also known as c-myc, is a multifunctional TF that plays a vital role in cell cycle progression, apoptosis, and cellular transformation^43^. *c-myc* expression is also up-regulated in ovarian endometriotic cyst stromal cells, and its expression can be suppressed by miR-196b^44^. The expression of c-MYC–regulated genes are associated with a higher risk for brain metastasis in breast cancer patients^45^. This paper demonstrates the different expressions of XLOC_l2_000941 in BM SCLC individuals.

*MYOM2*, a fourth high expression gene with Log FC = 2.60 in BM patients with the highest rate of missense mutations, is a protein-coding gene that usually plays a role in synthesising titin-associated protein fibronectin type III and immunoglobulin C2 domains^46^. Fibronectin is a cell adhesion protein that could affect metastasis^47^. *MYOM2* may associate with the pharmacology of Tumor necrosis factor (TNF) blockers^48^, a multifunctional cytokine that plays critical roles in diverse cellular events such as cell survival, proliferation, differentiation, and death alter cancer metastasis^49^. As we show in Table 2, *MYOM2* is associated with LINC01511 and LINC00448 lncRNAs. LINC01511 is up-regulated in triple-negative breast cancer and might play a fundamental role in the mechanism of EGFR exon 19 deletions in lung adenocarcinoma^50^.

*WHAMMP2* (WAS protein homolog associated with actin, Golgi membranes, and microtubules pseudogene 2) is a pseudogene highly expressed in BM SCLC similar to Thymocytes in Thymus in brain metastasis patients mentioned in table S4.

*TMEM176B* and *TMEM176A* have an extensive correlation with different lncRNAs, as shown in Table 2. Some of these lncRNAs get involved in cancers; for instance, *LINC00501* prohibits lung cancer development and metastasis by mediating miR-129-5p/HMGB1 and up-regulated in non-small cell lung cancer (NSCLC) patients^51^. Contrariwise, LBX2-AS1 promotes cell proliferation and metastasis through Notch signalling in NSCLC^52^. Also, we find that these two genes have physical interactions, co-expression relations, and shared protein domains, demonstrated in Fig. 3B. Transmembrane proteins (TMEMs) are membrane proteins with at least one transmembrane structure; They are also known as integral membrane proteins. *TMEM176* plays diverse functions in distinct malignancies. A range of membrane protein functions is intimately associated with the proliferation, invasion, and metastasis of malignant tumours^53,54^. In human hepatocellular carcinoma, epigenetic silencing of *TMEM176A* promotes ERK signalling^55^.

Similarly, high expression of *TMEM16A* plays a role in gastrointestinal stromal tumours^56^. *TMEM176A* in glioblastoma can inhibit *Bcl2* expression and cause apoptosis^57,58^. Increased *TMEM176B* protein levels have been found in various tumours, and inhibition of *TMEM176B* has been shown to increase CD8 + T cell-mediated tumour growth control, improving cancer therapy effectiveness^59^. *TMEM176B* represses adenosine triphosphate (ATP) and nigericin-induced NLRP3 inflammasome activation in dendritic cells and macrophages through ionic mechanisms. Targeting *TMEM176B* can emphasise anti-tumoral CD8^+^ T cell responses in an inflammasome-dependent manner while directly killing malignant cells^60,61^.

Phosphatidylinositol glycan anchor biosynthesis class C and W, respectively (*PIGC* and *PIGW*), are critical genes in the glycosylphosphatidylinositol (GPI)-anchor biosynthesis pathway, encode an endoplasmic reticulum (ER)-associated protein that is significant for the biosynthesis of glycosylphosphatidylinositol (GPI)^62^. This process happens in the endoplasmic reticulum and is a step in the glycosylphosphatidylinositol (GPI) biosynthesis, anchoring many cell surface proteins to the membrane. Defects in the *PIGW* gene cause the age-dependent epileptic encephalopathy West syndrome and a syndrome exhibiting hypophosphatasia and cognitive disability (HPMRS5)^63^. *PIGC* was up-regulated and associated with the transferase activity of cancer cells such as Hepatocellular carcinoma (HCC)^62^. Since cell adhesion can be altered in metastasis, it can indicate that in SCLC brain metastasis, GPI binding changes because of the high expression of *PIGC* and *PIGW*.

We indicate that *ABCA13*, an ATP-binding cassette (ABC) transporter, has the most mutations (9%) in lung cancer patients, as shown in Figure S2, Suggesting that alteration of this gene can affect the lung cancer state.

*AMFR* is a gene that encodes ubiquitin-protein ligase. It also correlates with lncRNAs, including FGF10-AS1, LINC02115, and LINC01716 (Table 2). Overexpression of gp78/AMFR in human cancers is related to low survival and the high stage of the tumours^64,65^. Thus, It can attribute narrow expression of AMFR in non-BM SCLC despite BM SCLC to a biomarker of this type of cancer metastasis. *CRAT* encodes an acetyltransferase and is involved in specific pathways among Beta-oxidation of very long-chain fatty acids, Beta-oxidation of pristanoyl-CoA, and Peroxisomal lipid metabolism (Table S3). CRAT is required to transport acetyl-CoA from peroxisomes or mitochondria into the cytoplasm to activate calcium/calmodulin-dependent kinase II (CaMKII). A decrease in CRAT protein levels reduces cell proliferation and low cell migration; it also is necessary for CaMKII activation^66^. *OCLN* is the most downregulated gene in DEGs results, with Log FC = − 3.56 (Table 1); naturally encoded transmembrane proteins play an essential role in regulating cell permeability barriers formed by protein tight junctions and Are an Integral Component of the blood–brain barrier (BBB)^67^.

We demonstrate that low expression of this gene may affect brain metastasis since prior studies reveal that CeRNA regulates OCLN mRNA, which plays a vital function in the BBB. PIK3CG is a component of the PI3K-Akt signalling pathway, controlling essential cell functions such as cell proliferation, differentiation, and metabolism^68,69^. *OCLN* also relates to three lncRNAs such as ARHGEF2-AS1, NKX2-1-AS1, and LINC01806 (Table 2). Two hub genes, including *CAT* and *APP,* are involved in different cancers; for Instance, CAT, positioned specially in peroxisomes, decomposes H_2_O_2_, a spinoff of fatty acid oxidation, to oxygen and water. Thus, CAT confers safety in opposition to the deadly consequences of H_2_O_2_ without producing intermediate unfastened radicals, and the ensuing oxygen is applied to different metabolic processes^70^. Catalase is likewise often downregulated in tumour tissues like non-small-cell lung cancer than in normal tissues of the exact origin^71^. H_2_O_2_ is a mediator of several physiological processes, including cell differentiation and proliferation, cellular metabolism, survival, and immune response, through ROS (reactive oxygen species) intracellular signalling (Fig. 1)^72^.

Amyloid precursor protein (APP) is a protein that has a role in the progression of Alzheimer's disease. Its cytoplasmic region contains numerous phosphorylation sites. APP has also been suggested as a molecule involved in cell proliferation and invasion in various human cancers, including non-small-cell lung cancer^73–75^. Our study reveals that these genes are downregulated In brain metastasis SCLC and suggests that they can influence the cancer stage and survival rate, as shown in Fig. 5. We suggest these genes mentioned above can be associated with brain metastasis in SCLC and might be employed as new therapeutic targets and potential prognosis biomarkers. However, considering these genes as a Biomarker require deep and comprehensive studies yet clinical validations. Since we performed only bioinformatical analysis, deficiencies still demand clinical experiments and data validation. Furthermore, these candidates' metastasis genes' functions and mechanisms are still to be investigated.

In conclusion, brain metastasis SCLC shows a different gene expression than non-BM SCLC. We demonstrated up and down-regulation of specific lncRNAs and mRNAs and their networks to evaluate their predictive values. Preventing cancer metastasis with reliable biomarkers can be vital; however, it is yet unknown to find the exact role of these distinct lncRNAs and how they influence cancer metastasis.

## Supplementary Information

Supplementary Information 1.

Supplementary Information 2.

## Data Availability

The expression data was gathered from Gene Expression Omnibus (GEO) (https://www.ncbi.nlm.nih.gov/geo/) (ACCESSION NUMBER: GSE161968) and enrich analysis performed utilising KEGG (https://www.genome.jp/kegg/), GO (http://geneontology.org/), and Kaplan–Meier survival analysis (https://kmplot.com/analysis/) databases. The analysed data and R codes are available to the corresponding author with no restriction upon request.
