# Supplementary material for: An in silico comparative transcriptome analysis identifying hub lncRNAs and mRNAs in brain metastatic small cell lung cancer (SCLC)
Source: Sci Rep. 2022 Oct 27;12:18063. doi: 10.1038/s41598-022-22252-7 (PMC9613661; doi:10.1038/s41598-022-22252-7)
Supplement: Supplementary file 1 — Supplementary Information 1. [file 41598_2022_22252_MOESM1_ESM.docx]

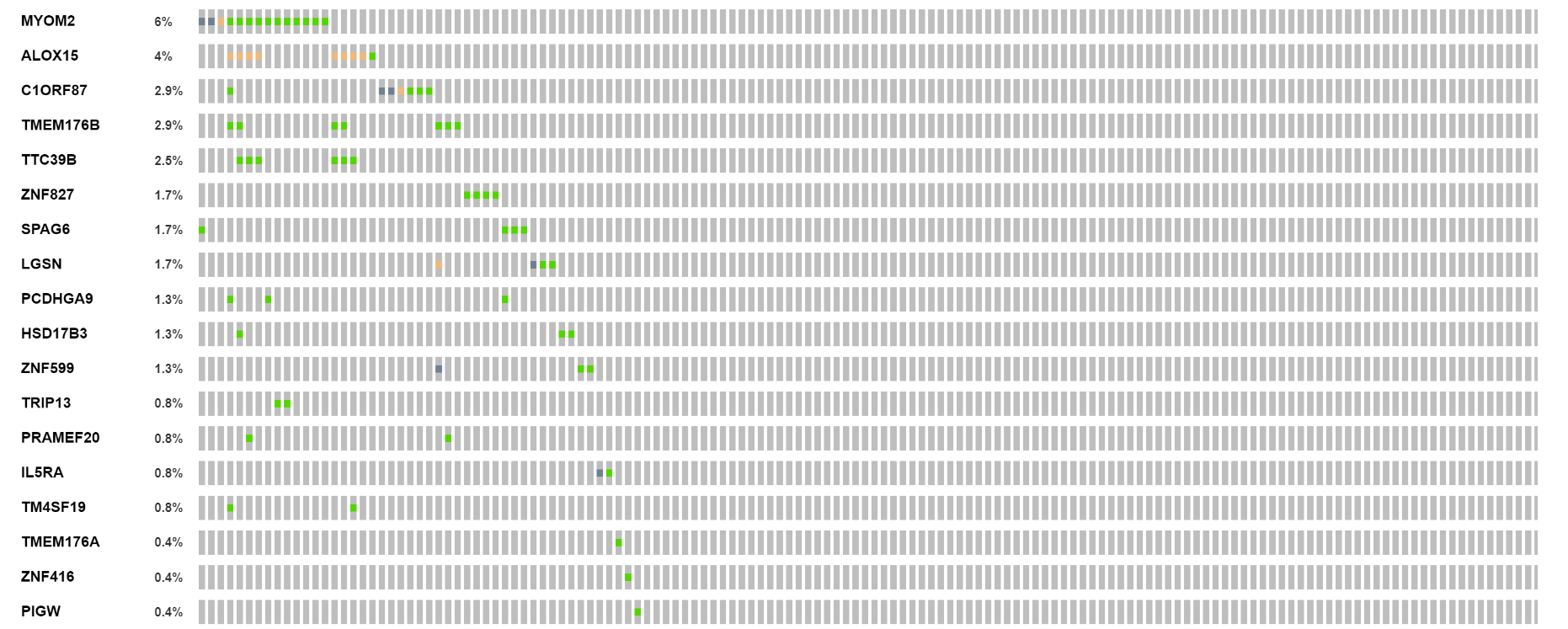

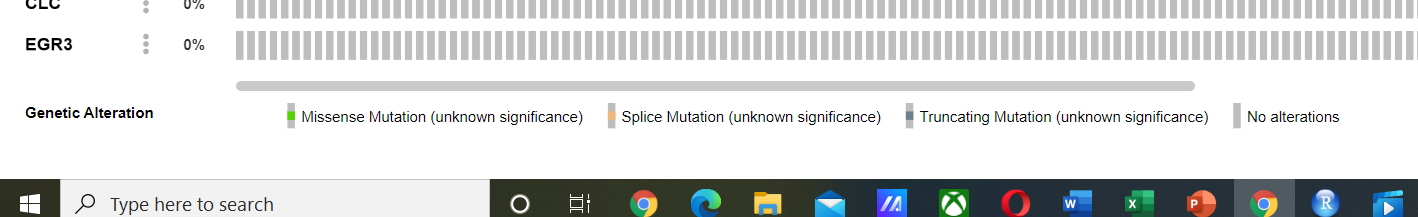


**Figure S1.** Mutations in up-regulated candidate genes in brain metastasis


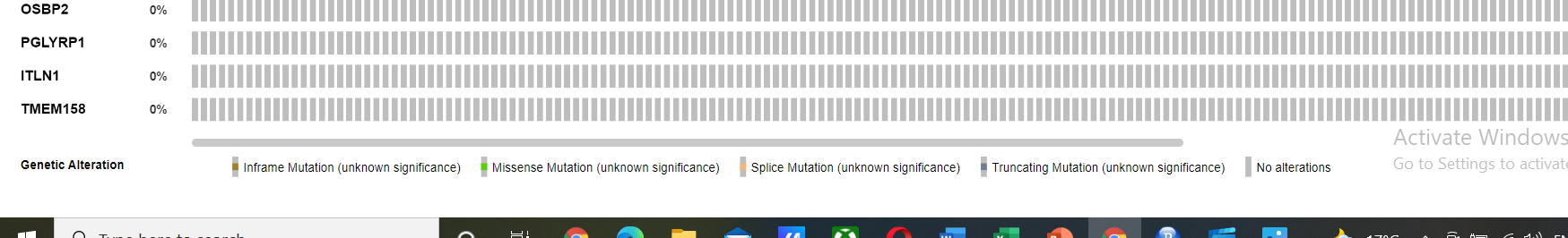

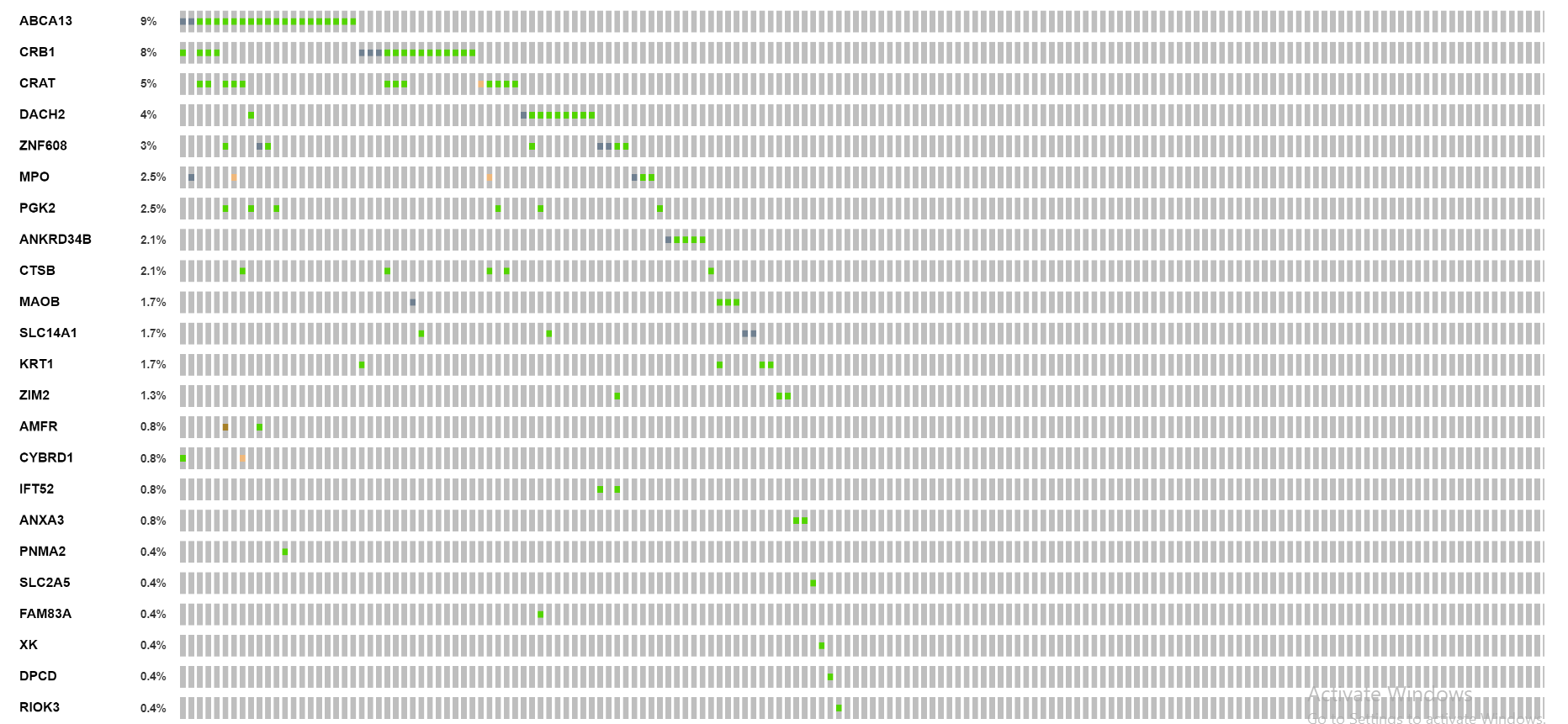


**Figure S2.**  Mutations in down-regulated candidate genes in brain metastasis
