# Supplementary material for: An in silico comparative transcriptome analysis identifying hub lncRNAs and mRNAs in brain metastatic small cell lung cancer (SCLC)
Source: Sci Rep. 2022 Oct 27;12:18063. doi: 10.1038/s41598-022-22252-7 (PMC9613661; doi:10.1038/s41598-022-22252-7)
Supplement: Supplementary file 2 — Supplementary Information 2. [file 41598_2022_22252_MOESM2_ESM.docx]

**Table S1.** KEGG (Kyoto Encyclopedia of Genes and Genomes) and GO (Gene Ontology) analysis report for both up and down-regulated Genes.

| KEGG | | | | | | |
| --- | --- | --- | --- | --- | --- | --- |
| Term | Overlap | P-value | Odds Ratio | Combined Score | Genes |  |
| Ferroptosis | 1/41 | 0.059748204247594700 | 17.18103448275860 | 48.409560113017700 | ALOX15 |  |
| Linoleic acid metabolism | 1/29 | 0.04262771511114430 | 24.559113300492600 | 77.49015815040230 | ALOX15 |  |
| The glycosylphosphatidylinositol (GPI)-anchor biosynthesis | 2/26 | 6.91239165097814E-04 | 59.36309523809520 | 431.9867091057640 | PIGC; PIGW |  |
| GO | | | | | |  |
| Term | Overlap | P-value | Odds Ratio | Combined Score | Genes |  |
| O-acyltransferase activity (GO:0008374) | 1/30 | 0.04406585504505750 | 23.711058263971500 | 74.02758505991470 | PIGW |  |
| lysophospholipase activity (GO:0004622) | 1/21 | 0.031047062800707400 | 34.39655172413790 | 119.43346355828800 | CLC |  |
| steroid dehydrogenase activity, acting on the CH-OH group of donors, NAD or NADP as acceptor (GO:0033764) | 1/21 | 0.031047062800707400 | 34.39655172413790 | 119.43346355828800 | HSD17B3 |  |
| oxidoreductase activity, acting on single donors with incorporation of molecular oxygen, incorporation of two atoms of oxygen (GO:0016702) | 1/18 | 0.02666950460061790 | 40.47261663286000 | 146.68225415832800 | ALOX15 |  |
| cysteine-type endopeptidase activity involved in the apoptotic process (GO:0097153) | 1/15 | 0.022272833024450600 | 49.152709359605900 | 186.9959576603660 | CLC |  |

**Table S2.** Top pathways considered to up-regulated genes in brain metastasis patients (BioPlanet 2019 database)

| Key pathways | P-value | Genes |
| --- | --- | --- |
| Inhibition of activated T cell apoptosis by neuropeptides VIP and PACAP | 0.038301 | EGR3 |
| Interleukin-5 regulation of apoptosis | 0.019628 | EGR3; IL5RA |
| Androgen biosynthesis | 0.014902 | HSD17B3 |
| Steroid biosynthesis | 0.038301 | HSD17B3 |
| Interleukin-3 regulation of hematopoietic cells | 0.02959 | IL5RA |
| Interleukin-9 regulation of target genes | 0.038301 | IL5RA |
| Interleukin receptor SHC signaling | 0.041187 | IL5RA |
| Inflammatory response pathway | 0.044066 | IL5RA |
| Lysosphingolipid and lysophosphatidic acid (LPA) G-protein coupled receptors | 0.011939 | LPAR3 |
| Stress fiber formation mediated by Rho-selective guanine exchange factor AKAP13 | 0.016381 | LPAR3 |
| Glycosylphosphatidylinositol (GPI) biosynthesis | 2.92E-04 | PIGC; PIGW |
| Post-translational modification: biosynthesis of GPI-anchored proteins | 8.61E-04 | PIGC; PIGW |
| Post-translational protein modification | 0.034728 | PIGC; PIGW |

**Table S3.** Critical pathways Involved In down-regulated genes in brain metastasis patients (BioPlanet 2019 database)

| Key pathways | P-value | Genes |
| --- | --- | --- |
| Prostaglandin biosynthesis and regulation | 0.046983597 | ANXA3 |
| Oncostatin M | 0.012132358 | ANXA3; KRT1; CTSB |
| Beta-oxidation of very-long-chain fatty acids | 0.009265092 | CRAT |
| Beta-oxidation of pristanoyl-CoA | 0.012334969 | CRAT |
| Peroxisomal lipid metabolism | 0.03056186 | CRAT |
| Toll-like receptor endosomal trafficking and processing | 0.018447136 | CTSB |
| Corticotropin-releasing hormone pathway | 0.038059951 | KRT1 |
| Phenylalanine metabolism | 0.026035892 | MAOB |
| Histidine metabolism | 0.044017987 | MAOB |
| Glycine, serine, and threonine metabolism | 0.048463062 | MAOB |
| Alpha-synuclein signalling | 0.048463062 | MAOB |
| Apoptotic cleavage of cell adhesion proteins | 0.016922535 | OCLN |
| Toll receptor cascades | 0.025116345 | PGLYRP1; CTSB |
| Amine compound SLC transporters | 0.044017987 | SLC14A1 |
| Transmembrane transport of small molecules | 0.028760625 | SLC14A1; CYBRD1; SLC2A5 |
| Transport of glucose and other sugars, bile salts and organic acids, metal ions, and amine compounds | 0.009684437 | SLC14A1; SLC2A5 |
| Facilitative sodium-independent glucose transporters | 0.018447136 | SLC2A5 |
| Influenza factor interactions with host | 0.009265092 | XK |

**Table S4****.** Key cell types and tissues related to up and down-regulated genes in brain metastasis patients (Descartes Cell Types and Tissue 2021 database)

| Key cell types and tissues | P-value | Up Genes | Key cell types and tissues | P-value | Down Genes |
| --- | --- | --- | --- | --- | --- |
| Excitatory neurons in Cerebrum | 0.031047 | ALOX15 | Epicardial fat cells in the Heart | 0.012346 | ITLN1; FAM83A |
| Megakaryocytes in Spleen | 0.019628 | EGR3; TTC39B | Hematopoietic stem cells in the Liver | 0.04994 | MPO |
| CLC IL5RA positive cells in the Heart | 0.002858 | IL5RA; CLC | Erythroblasts in Adrenal | 5.84E-04 | SLC14A1; KRT1; ITLN1 |
| Astrocytes in Cerebrum | 0.032809 | SPAG6; IL5RA | Erythroblasts in Kidney | 0.003603 | SLC14A1; KRT1; OSBP2 |
| Ciliated epithelial cells in Lung | 7.43E-04 | SPAG6; LGSN; IL5RA; LPAR3; TRIP13 | Erythroblasts in Liver | 0.005859 | SLC14A1; OSBP2 |
| Schwann cells in Adrenal | 0.001001 | TMEM176B; ALOX15; TMEM176A | Erythroblasts in Stomach | 0.01367 | SLC14A1; OSBP2 |
| Thymocytes in Thymus | 0.022328 | WHAMMP2; TM4SF19; MYOM2 | Erythroblasts in Muscle | 0.035192 | SLC14A1; XK |
|  | | | Erythroblasts in Pancreas | 6.25E-05 | SLC14A1; XK; KRT1; OSBP2 |
|  |  |  | Erythroblasts in Intestine | 0.002893 | SLC14A1; XK; OSBP2 |

**Table S5.** protein classes for up-regulated genes in BM SCLC patients (Panther

database)

| Mapped IDs | PANTHER Protein Class |
| --- | --- |
| PIGW | Acyltransferase (PC00042) |
| ZNF827 | C2H2 zinc finger transcription factor (PC00248) |
| ZNF599 | C2H2 zinc finger transcription factor (PC00248) |
| EGR3 | C2H2 zinc finger transcription factor (PC00248) |
| PCDHGA9 | Cadherin (PC00057) |
| TRIP13 | chromatin/chromatin-binding, or -regulatory protein (PC00077) |
| CLC | extracellular matrix protein (PC00102) |
| PIGC | Glycosyltransferase (PC00111) |
| LPAR3 | G-protein coupled receptor (PC00021) |
| EMR4P | G-protein coupled receptor (PC00021) |
| SIGLEC8 | immunoglobulin superfamily cell adhesion molecule (PC00125) |
| LGSN | Ligase (PC00142) |
| SPAG6 | microtubule or microtubule-binding cytoskeletal protein (PC00157) |
| ALOX15 | Oxygenase (PC00177) |
| PRSS33 | serine protease (PC00203) |
| IL5RA | transmembrane signal receptor (PC00197) |

**Table S6.** protein classes for down-regulated genes in BM SCLC patients (Panther database)

| Mapped IDs | PANTHER Protein Class |
| --- | --- |
| CRAT | Acyltransferase (PC00042) |
| ABCA13 | ATP-binding cassette (ABC) transporter (PC00003) |
| ZIM2 | C2H2 zinc finger transcription factor (PC00248) |
| ANXA3 | calcium-binding protein (PC00060) |
| CRB1 | cell adhesion molecule (PC00069) |
| CTSB | cysteine protease (PC00081) |
| OCLN | general transcription factor (PC00259) |
| RIOK3 | non-receptor serine/threonine-protein kinase (PC00167) |
| MAOB | Oxidase (PC00175) |
| MPO | Peroxidase (PC00180) |
| CYBRD1 | Reductase (PC00198) |
| SLC14A1 | Transporter (PC00227) |
| AMFR | ubiquitin-protein ligase (PC00234) |
| DACH2 | winged helix/forkhead transcription factor (PC00246) |

**Table S7.1.** Key Transcription factors for candidates up and down-regulated genes in brain metastasis patients (TRUST database 2019)

| Key TF | P-value | Up Genes | Key TF | P-value | Down Genes |
| --- | --- | --- | --- | --- | --- |
| SOX5 mouse | 0.0089673 | SPAG6 | **ETS1 human** | 0.006651 | TMEM158; CTSB |
| NFATC1 human | 0.0104543 | EGR3 | **HEY1 human** | 0.010801 | OCLN |
| NFATC2 human | 0.0104543 | EGR3 | **SP3 human** | 0.013222 | MAOB; CTSB |
| TCF3 human | 0.020803 | IL5RA | **SNAI2 mouse** | 0.015396 | OCLN |
| RFX1 human | 0.0222728 | IL5RA | **SP4 human** | 0.016923 | MAOB |
| POU2F2 mouse | 0.0252061 | IL5RA | **RXRA mouse** | 0.023007 | MPO |
| NFATC2 mouse | 0.0310471 | EGR3 | **SP1 human** | 0.036014 | MAOB; MPO; CTSB |
| MTA1 human | 0.0354056 | ALOX15 | **RARA human** | 0.039553 | MAOB |
| CREBBP human | 0.0368542 | ALOX15 | **SNAI1 mouse** | 0.044018 | OCLN |
| CREM human | 0.0368542 | IL5RA |  |  |  |
| MEF2C mouse | 0.0411875 | MYOM2 |  |  |  |

**Table S7.2.** Key miRNAs related to up and down-regulated genes in brain metastasis patients (miRTarBase_2017 database)

| miRNA | P-value | Up Genes | miRNA | P-value | Down Genes |
| --- | --- | --- | --- | --- | --- |
| hsa-miR-219b-3p | 0.037686267 | ALOX15; PIGW | **hsa-miR-4759** | 0.048463 | AMFR |
| hsa-miR-302f | 0.045307516 | ALOX15; PIGW | **hsa-miR-505-3p** | 0.046198 | AMFR; PNMA2 |
| hsa-miR-3941 | 0.025919592 | EGR3; IL5RA; LPAR3 | **hsa-miR-3664-3p** | 0.030298 | AMFR; SLC2A5 |
| hsa-miR-603 | 0.039820521 | EGR3; IL5RA; LPAR3 | **hsa-miR-140-3p** | 0.046947 | AMFR; SLC2A5 |
| hsa-miR-362-3p | 0.045503499 | EGR3; IL5RA; LPAR3 | **mmu-miR-5046** | 0.010801 | ANKRD34B |
| hsa-miR-329-3p | 0.045924305 | EGR3; IL5RA; LPAR3 | **mmu-miR-5106** | 0.019969 | ANKRD34B |
| hsa-miR-873-3p | 0.02334179 | EGR3; SIGLEC8 | **mmu-miR-574-5p** | 0.042532 | CTSB |
| mmu-miR-92b-3p | 0.019331061 | IL5RA | **hsa-miR-1247-5p** | 0.030562 | CYBRD1 |
| mmu-miR-367-3p | 0.020803015 | IL5RA | **mmu-miR-323-5p** | 0.042532 | CYBRD1 |
| mmu-miR-153-3p | 0.020803015 | IL5RA | **hsa-miR-5090** | 0.04994 | FAM83A |
| mmu-miR-32-5p | 0.020803015 | IL5RA | **hsa-miR-554** | 0.033568 | MAOB |
| mmu-miR-25-3p | 0.022272833 | IL5RA | **hsa-miR-1295a** | 0.045502 | OCLN |
| mmu-miR-363-3p | 0.022272833 | IL5RA | **mmu-miR-339-5p** | 0.042884 | OCLN; CYBRD1 |
| mmu-miR-92a-3p | 0.031047063 | IL5RA | **mmu-miR-10b-5p** | 0.044713 | OCLN; CYBRD1 |
| mmu-miR-669i | 0.038300736 | IL5RA | **mmu-miR-10a-5p** | 0.045083 | OCLN; CYBRD1 |
| mmu-miR-669j | 0.038300736 | IL5RA | **hsa-miR-6124** | 0.031897 | OCLN; MAOB |
| mmu-miR-448-3p | 0.039745158 | IL5RA | **mmu-miR-324-3p** | 0.015036 | OCLN; RIOK3; CRAT |
| mmu-miR-1958 | 0.046935873 | IL5RA | **mmu-miR-706** | 0.006998 | OCLN; RIOK3; CYBRD1 |
| hsa-miR-1911-5p | 0.048367756 | LGSN | **mmu-miR-3108-5p** | 0.039553 | OSBP2 |
| hsa-miR-378i | 0.04979756 | LGSN | **mmu-miR-300-3p** | 0.009265 | PNMA2 |
| hsa-miR-378f | 0.04979756 | LGSN | **hsa-miR-7854-3p** | 0.023101 | PNMA2; CTSB |
| hsa-miR-378h | 0.04979756 | LGSN | **mmu-miR-694** | 0.006815 | RIOK3; OSBP2 |
| hsa-miR-378e | 0.04979756 | LGSN | **mmu-miR-669f-5p** | 0.015396 | XK |
| hsa-miR-378d | 0.04979756 | LGSN | **mmu-miR-669a-5p** | 0.015396 | XK |
| hsa-miR-1273e | 0.030823915 | LGSN; ALOX15; PIGW | **mmu-miR-669l-5p** | 0.015396 | XK |
| hsa-miR-4257 | 0.019032165 | LGSN; MYOM2; TTC39B | **mmu-miR-669p-5p** | 0.015396 | XK |
| hsa-miR-2467-3p | 0.027826003 | LGSN; MYOM2; TTC39B | **mmu-miR-669h-3p** | 0.045502 | XK |
| hsa-miR-6086 | 0.008519386 | LGSN; PIGW; MYOM2; TTC39B | **mmu-miR-669k-3p** | 0.045502 | XK |
| hsa-miR-377-5p | 0.009877889 | LGSN; PIGW; MYOM2; TTC39B | **mmu-miR-804** | 0.043248 | XK; RIOK3 |
| hsa-miR-1200 | 0.023894276 | LGSN; SIGLEC8 | **hsa-miR-29c-3p** | 0.006998 | XK; RIOK3; AMFR |
| hsa-miR-4324 | 0.027321238 | LGSN; SIGLEC8 | **mmu-miR-190b-5p** | 0.009265 | ZNF608 |
| hsa-miR-544b | 0.003418152 | LGSN; SIGLEC8; MYOM2 | **mmu-miR-669f-3p** | 0.040728 | ZNF608; OSBP2 |
| hsa-miR-4318 | 0.004266289 | LGSN; TRIP13 |  |  |  |
| mmu-miR-384-5p | 0.04979756 | LPAR3 |  |  |  |
| mmu-miR-2136 | 0.020803015 | TMEM176B |  |  |  |
| hsa-miR-7706 | 0.020803015 | TRIP13 |  |  |  |
| hsa-miR-4800-5p | 0.045501907 | TRIP13 |  |  |  |
| mmu-miR-3970 | 0.014902355 | TTC39B |  |  |  |
| mmu-miR-19a-3p | 0.039745158 | TTC39B |  |  |  |
| hsa-miR-6880-3p | 0.032502015 | ZNF416 |  |  |  |
| hsa-miR-4643 | 0.028505412 | ZNF827; LPAR3 |  |  |  |

**Table S8.1.** Key histone modifications related to up-regulated genes in brain metastasis patients (ENCODE Histone Modifications database)

| histone modifications | P-value | Genes |
| --- | --- | --- |
| H3K27me3 kidney epithelial cell hg19 | 0.01409 | SPAG6; TMEM176B; ALOX15; TMEM176A; LPAR3; C1ORF87; PRSS33; MYOM2 |
| H3K9me3 fibroblast of dermis hg19 | 0.02573 | PIGC; SPAG6; EMR4P; IL5RA; C1ORF87; PIGW; PCDHGA9 |
| H3K9me3 mammary epithelial cell hg19 | 0.02573 | SPAG6; EMR4P; ZNF416; IL5RA; ZNF599; SIGLEC8; PCDHGA9 |
| H3K27me3 fibroblast of lung hg19 | 0.03061 | SPAG6; TMEM176B; ALOX15; TMEM176A; IL5RA; LPAR3; C1ORF87; CLC; PRSS33 |
| H3K27me3 bronchial epithelial cell hg19 | 0.03127 | EGR3; SPAG6; TMEM176B; ALOX15; TMEM176A; C1ORF87; PRSS33 |
| H3K9me3 MCF-7 hg19 | 0.03281 | ZNF599; PCDHGA9 |
| H3K27me3 mammary epithelial cell hg19 | 0.03284 | SPAG6; LGSN; TMEM176B; ALOX15; TMEM176A; C1ORF87; PRSS33; MYOM2 |
| H3K27me3 keratinocyte hg19 | 0.04246 | SPAG6; TMEM176B; TMEM176A; IL5RA; CLC; MYOM2; PCDHGA9 |
| H3K27me3 K562 hg19 | 0.04917 | ZNF827; EMR4P; ALOX15; IL5RA; LPAR3; TRIP13; CLC; MYOM2; PCDHGA9 |

**Table S8.2.** Key histone modifications related to down-regulated genes in brain metastasis patients (ENCODE Histone Modifications database)

| histone modifications | P-value | Genes |
| --- | --- | --- |
| H3K27me3 astrocyte hg19 | 0.030453 | ANKRD34B; OCLN; KRT1; PNMA2; DACH2; PGLYRP1; OSBP2 |
| H3K27me3 cardiac mesoderm hg19 | 0.032817 | ANKRD34B; OCLN; XK; TMEM158; MAOB; ANXA3; CYBRD1; PNMA2; CRAT; OSBP2 |
| H3K27me3 keratinocyte hg19 | 0.005236 | ANKRD34B; OCLN; ZNF608; PNMA2; DACH2; PGLYRP1; MPO; ZIM2; OSBP2 |
| H3K27me3 CD14-positive monocyte hg19 | 0.030526 | ANKRD34B; OCLN; ZNF608; XK; MAOB; ANXA3; PNMA2; PGLYRP1; ZIM2; FAM83A |
| H3K4me1 keratinocyte hg19 | 0.030453 | ANKRD34B; SLC14A1; OCLN; CYBRD1; DACH2; ABCA13; CRAT |
| H3K27me3 MCF-7 hg19 | 0.003661 | CRB1; ZNF608; TMEM158; KRT1; CYBRD1; DACH2 |
| H3K4me1 erythroblast mm9 | 0.0049 | IFT52; OCLN; TMEM158; ANXA3; CYBRD1; PNMA2; DACH2; PGLYRP1; DPCD; OSBP2 |
| H3K27me3 CH12.LX mm9 | 0.041992 | OCLN; TMEM158; ITLN1; PGLYRP1; CRAT; FAM83A; CTSB; OSBP2 |
| H3K4me1 HCT116 hg19 | 0.030453 | ZNF608; XK; MAOB; ANXA3; RIOK3; CYBRD1; FAM83A |

**Table S9.** Top 10 diseases related to up and down-regulated genes in brain metastasis patients (PheWeb 2019 database)

| Key diseases | P-value | Up Genes | Key diseases | P-value | Down Genes |
| --- | --- | --- | --- | --- | --- |
| Stricture/obstruction of the ureter | 5.88E-04 | IL5RA; TTC39B | Inflammatory diseases of the uterus, except cervix | 0.010801 | ZIM2 |
| Somatoform disorder | 0.023741 | TTC39B | Schmorl's nodes | 0.015396 | ZNF608 |
| Portal hypertension | 0.025206 | TMEM176B | Spontaneous ecchymoses | 0.016923 | ZIM2 |
| Fracture of unspecified part of the femur | 0.028131 | IL5RA | Neoplasm of uncertain behaviour of breast | 0.018447 | DACH2 |
| Disorders involving the immune mechanism | 0.028131 | LPAR3 | Non-proliferative glomerulonephritis | 0.018447 | MAOB |
| Postinflammatory pulmonary fibrosis | 0.02959 | HSD17B3 | Circadian rhythm sleep disorder | 0.019969 | ABCA13 |
| Premature beats | 0.031047 | LGSN | Scar conditions and fibrosis of the skin | 0.019969 | DACH2 |
| Missed abortion/Hydatidiform mole | 0.032502 | LPAR3 | Sacroiliitis NEC | 0.021489 | ZNF608 |
| Urinary incontinence | 0.033955 | LPAR3 | Chromosomal anomalies and genetic disorders | 0.021489 | ZNF608 |
| Carditis | 0.033955 | ZNF827 | Adrenal cortical steroids cause adverse effects in therapeutic use | 0.021489 | ZNF608 |
